# Effects of Acute Guarana (*Paullinia cupana*) Ingestion on Mental Performance and Vagal Modulation Compared to a Low Dose of Caffeine

**DOI:** 10.3390/nu16121892

**Published:** 2024-06-15

**Authors:** Tyler N. Talik, Eduardo Macedo Penna, Brian P. Hack, Alec Harp, Mindy Millard-Stafford

**Affiliations:** 1 School of Biological Sciences, Georgia Institute of Technology, Atlanta, GA 30318, USA; tylertpro1@gmail.com (T.N.T.); bhack@gatech.edu (B.P.H.); aharp6@gatech.edu (A.H.); 2 Physical Education Faculty, Federal University of Pará, Castanhal 68746-630, PA, Brazil; eduardomp@ufpa.br

**Keywords:** cognition, mood, fatigue, perception, emotion, HRV, vagus nerve

## Abstract

Guarana (GUA), a Brazilian seed extract, contains caffeine and other bioactive compounds that may have psychoactive effects. To assess the acute effects of GUA compared to a low dose of caffeine (CAF) on cognitive and mood parameters, twenty participants completed a double-blind, crossover experiment where they ingested capsules containing the following: (1) 100 mg CAF, (2) 500 mg GUA containing 130 mg caffeine, or (3) placebo (PLA). Cognitive tests (Simon and 2N-Back Task) were performed at the baseline (pre-ingestion) and 60 min after ingestion. The response time for the cognitive tests and heart rate variability were unaffected (*p* > 0.05) by treatment, although 2N-Back was overall faster (*p* = 0.001) across time. The accuracy in the 2N-Back Task showed a significant interaction effect (*p* = 0.029) due to higher post-ingestion versus pre-ingestion levels (*p* = 0.033), but only with the PLA. The supplements also had no effect on cognitive measures following physical fatigue (*n* = 11). There was an interaction effect on perceived mental energy, where the pre-ingestion of GUA had lower mental pep ratings compared to post-ingestion (*p* = 0.006) and post-exercise (*p* = 0.018) levels. Neither the acute ingestion of GUA nor low dose of CAF influenced cognitive performance or provided consistent benefit on mood or mental workload through vagal modulation. Additional investigations are beneficial to determining the lowest effective dose for CAF or GUA to influence mood and/or cognitive performance.

## 1. Introduction

Mental performance is characterized by the effectiveness of an individual’s cognitive function to accomplish tasks [1]. Mood, feelings, and emotions may also influence mental performance [2], specifically mental fatigue which is a cause of impaired attention and reduced accuracy [3,4]. To combat mental fatigue and enhance cognitive functions, strategies such as exercise and nutritional aids have been suggested [5,6]. Among these, caffeine stands out as the most popular and extensively used stimulant, with over 90% of adults in the United States consuming it regularly [7]. Acute caffeine ingestion significantly improves various cognitive and mood-related variables, including response time, attention, and mental fatigue [8]. The beneficial effects of caffeine are primarily attributed to its role as an adenosine receptor antagonist in the brain [9], blocking adenosine from binding to the receptors and activating them [10], thereby inducing the sympathetic physiological responses of wakefulness, alertness, and arousal [10]. Furthermore, this activation may also lead to physiological changes such as increased blood pressure and heightened muscular activity [11,12].

Factors such as daily consumption habits, age, gender, body size, medical conditions, and genetics [13] all influence individual mood and cognitive responses to caffeine doses [14]. Habitual caffeine consumers often experience more significant cognitive improvements, especially in spatial memory and rapid visual information processing, than non-habitual drinkers [15]. Moreover, the effective caffeine dosage for acute cognitive benefits is debated but also generally observed at 200 mg or higher [16], although smaller doses (below 3 mg per kilogram of body weight) may enhance performance in attention-related tasks [17]. While black coffee is the most common source of caffeine, Guarana, an Amazonian rainforest plant, has emerged with potential cognitive and mood benefits [18]. Guarana seed extracts, depending on the source and processing, have a caffeine content of 5% to 10% [18,19], 0.9% to 2.4% higher than coffee beans [20]. A few studies have indicated that the low natural dose of caffeine, commonly found in Guarana, can separately enhance memory, vigilance, alertness, and information processing speed [21,22]. Whether the effect of Guarana is due solely to its caffeine content for cognitive effects remains unclear.

Guarana also contains bioactive compounds (e.g., flavonoids, methylxanthines, and proanthocyanidins), which may exert certain effects [23,24]. However, few studies have systematically controlled the impact of caffeine versus matched caffeine levels in Guarana. Faster response times (RT) in a Go/No-go Task were found with Guarana (GUA) compared to caffeine (CAF) [25]; but in another study, no differences were found between GUA and CAF for RT during a Simon Task [26]. Furthermore, a meta-analysis of eight placebo-controlled studies indicated that GUA slightly improves RT in cognitive tests without affecting performance accuracy [27]. Moreover, other individual studies have revealed the potential of GUA to enhance alertness, secondary memory accuracy, and working memory, as measured by different cognitive and mood assessments [28,29]. However, not all studies consistently support the effect of Guarana, when compared to the placebo, on cognitive function, underscoring the need for further investigation into its specific effects and the role that its caffeine content may play.

The knowledge about the effects of Guarana to counter fatigue has wide applications, including enhancing athletic performance. Mental fatigue has been shown to reduce endurance, sprint, and jump performance in athletes [30,31]. Caffeine has been recognized to improve various exercise performance metrics [32]. In comparison, limited research has compared Guarana’s effect on cognitive performance and mood to that of caffeine, especially in situations eliciting mental or physical fatigue. Investigations of Guarana have mainly focused on working memory and response inhibition after exhaustive cycling [33] or running [34] with unmatched doses of CAF, leaving a knowledge gap in the benefits of GUA over CAF following physical and/or mental fatigue. Whether the caffeine level naturally found in GUA is primarily responsible for the benefits and if a low dose of caffeine (either in GUA or caffeine by itself) improves mental performance remains unclear.

Therefore, we had the following two questions: (1) Does a low dose of caffeine elicit mental performance benefits? And (2) if so, would Guarana perform similarly when the caffeine dose available in the supplement is similarly matched? The acute effects of GUA ingestion on mood (Brunel Mood Scale), cognitive performance (2N-Back and Simon Tasks), motivation/mental energy (Motivation and Energy Visual Analog Scales), and mental fatigue (NASA Task Load) were compared to a low dose of CAF and a placebo. In addition, the effect of GUA following physical fatigue and the role of sympathetic activation as a mechanism for these effects was analyzed. We hypothesized that GUA would improve both mood and performance on cognitive tasks without added mental fatigue compared to the placebo control but elicit similar effects to a low dosage of CAF.

## 2. Materials and Methods

### 2.1. Experimental Design

**Figure 1:**
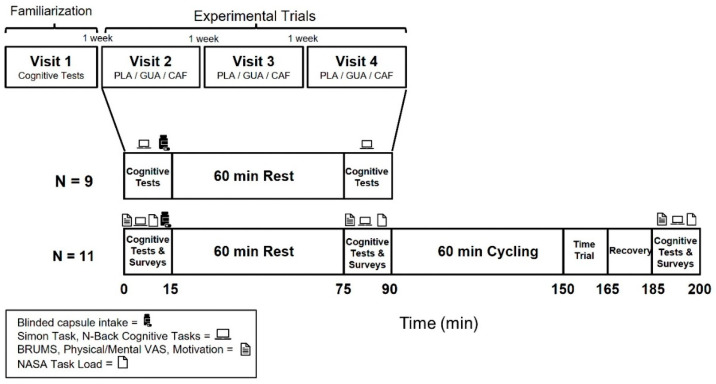
Schematic of the test protocol. CAF = Caffeine; GUA = Guarana; PLA = Placebo. BRUMS, VAS, NASA are survey instruments described in methods.

The study utilized a double-blind, placebo-controlled crossover with a Latin square block design for treatment assignments. Participants visited the laboratory four times (once for preliminary testing/familiarization with procedures followed by three experimental trials), each separated by at least one week, occurring at the same hour of day within each participant. A schematic of the test protocol is shown (Figure 1).

### 2.2. Participants

Twenty participants (healthy adult volunteers) were recruited at two locations, one in Brazil and the other in the United States. The selection of participants was a convenient sampling of willing volunteers (non-compensated) available to the research group. All provided written consent as approved by the local Institutional Review Boards. All procedures were conducted according to the Declaration of Helsinki. Participants were requested to avoid any caffeine consumption and vigorous training 24 h before the experimental sessions. The participants’ physical characteristics (17 men, 3 women) were as follows: age = 22.5 ± 4.2 y, height = 1.79 ± 0.07 m, body mass = 70.3 ± 11.1 kg). Eleven athlete participants were recruited to perform the cognitive testing protocol, followed by a cycling exercise to elicit physical fatigue. For the exercise subgroup, average caffeine intake was estimated to be 131 ± 78 mg/day. Habitual intake ranged from none (two participants) to ~250 mg (two participants listed two to three cups of coffee per day).

### 2.3. Interventions

#### 2.3.1. Preliminary Testing and Familiarization

Participants were informed about all the procedures during the first laboratory visit and familiarized with the Simon and 2N-back cognitive tasks. In addition to brief written instructions provided by the task program, verbal instructions for both tasks were given by researchers to participants. Participants were then given as many attempts as needed to learn the tasks with practice versions of each task that included computerized feedback on accuracy. Familiarization with each cognitive test was complete when the participants indicated that they felt comfortable after performing the experimental versions of the Simon and 2N-Back Tasks. Mood and fatigue surveys were verbally explained by researchers and practiced once by only the exercise subgroup.

In the exercise subgroup, a ramp test was performed to measure endurance capacity on an electronically braked cycle ergometer (Lode Excalibur, Groningen, The Netherlands) to set workloads at moderate intensity during the experimental sessions as described previously [35].

#### 2.3.2. Supplement Administration

The CAF, GUA, and PLA treatments were administered orally in a capsule form with 50 mL of tap water, while the participants were blindfolded. Since the capsules were selected and coded by a team member not involved in the data collection, the researchers were blinded. The exercise subgroup was asked after each experimental session about which substance they thought had been ingested, but none were able to correctly identify any capsule.

The same absolute low dose of CAF and GUA dose was administered (not adjusted relative to participants’ body mass). The CAF capsules contained 100 mg caffeine anhydrous, while the GUA capsules contained 500 mg GUA (with 100 mg caffeine listed on the label), purchased from the same manufacturer (Nutricost, Vineyard, UT, USA). Following the conclusion of the experiment, independent verification (North American Lab, National Sanitation Foundation [NSF], Ann Arbor, MI, USA) of CAF content was obtained. The NSF found that GUA had higher caffeine content (130 mg) compared with the label (100 mg). Based on the participants’ body mass (BM), the caffeine dosage averaged 1.45 ± 0.24 (1.14–1.98) and 1.89 ± 0.31 (1.48–2.58) mg/kg BM for the CAF and GUA treatments, respectively, both of which are relatively low doses of caffeine.

#### 2.3.3. Cognitive and Mood Assessments Administration

All participants arrived at the laboratory at approximately the same time in the morning following an overnight fast. The baseline cognitive tests were performed in approximately 10 min for all the participants, directly before and 60 min after capsule ingestion, under seated resting conditions in a thermally neutral controlled laboratory (23 °C, 40% RH). The computerized cognitive tasks were the Simon Task and the 2N-Back Task. For the exercise subgroup, mood scales and surveys were also completed directly after the cognitive tests and 20 min after the completion of a fatigue-inducing 75 min cycling bout.

### 2.4. Experimental Cognitive and Mood Assessments

#### 2.4.1. Simon Task

The Simon Task was programmed using PsychoPy. The Simon Task is commonly used to assess response inhibition and attention [36]. Participants are instructed to press the left arrow key for a red square and right arrow key for a blue square, while ignoring the position of the square on the screen (left or right), while responding as quickly and accurately as possible. The primary outcome measures were accuracy (ACC) and response time (RT). ACC was the percentage of correct responses out of a total 80 responses possible. RT was the time from stimulus presentation until an arrow key was pressed.

#### 2.4.2. 2N-Back Task

The 2N-Back Task was also developed on PsychoPy. The 2N-Back Task is a commonly used cognitive task to assess working memory [37]. Participants viewed a continuous string of letters (A–F) and indicated whether the current letter matched the letter shown two letters prior. The primary outcome measures were ACC and RT. ACC was expressed as a percentage of the total correct responses, in which the space key was pressed corresponding to a letter match. A total of 80 responses were collected. RT was measured as the time from the correctly matched letter stimuli presentation to space key press.

#### 2.4.3. The Brunel Mood Scale

The Brunel Mood scale (BRUMS) is a self-reported survey with 24 item ratings using a five-point Likert scale [38]. The subscales (anger, confusion, depression, fatigue, tension, and vigor) are derived from the sum of corresponding item ratings in the range of 0–16, with a higher score denoting a higher feeling for that particular mood attribute.

#### 2.4.4. The NASA Task Load Index

The NASA Task Load Index is a self-reported survey with six questions [39] corresponding to the perceived level of mental workload, with higher ratings reflecting a higher perception of that type of mental workload. The workload types include mental demand, physical demand, temporal demand, performance, effort, and frustration.

#### 2.4.5. Motivation and Energy Visual Analog Scales

The Motivation and Energy Visual Analog Scales is a self-reported survey with seven questions and corresponding items [40]. The mental and physical activities items include energy, fatigue, vigor, exhaustion, pep, worn out, and an overall motivation.

### 2.5. Experimental Physiological Assessments

#### Heart Rate Variability

Heart rate variability (HRV) was recorded for five minutes at the starting points of pre-ingestion, post-ingestion, and post-exercise in the exercise subgroup using a chest monitor (H10, Polar) connected to the HRV Elite APP, previously validated in another study [41]. HRV was recorded using the root mean square of successive differences between normal heartbeats (RMSSD). Increased resting RMSSD is a potential indicator of psychological resiliency and flexibility, indicating an increased ability to effectively adapt to altering demands and stressors [42].

### 2.6. Statistical Analysis

Analyses were performed using IBM SPSS software (version 29). A two-factor analysis of variance with repeated measures (supplement × time) was utilized to determine differences among treatments at baseline (prior to ingestion), post-ingestion, and post-exercise (in the exercise sub-group). If a significant difference was observed, pairwise comparisons were analyzed with Bonferroni post hoc tests. Statistical significance was denoted by an alpha level of *p* < 0.05.

## 3. Results

### 3.1. Simon Task Performance

**Figure 2:**
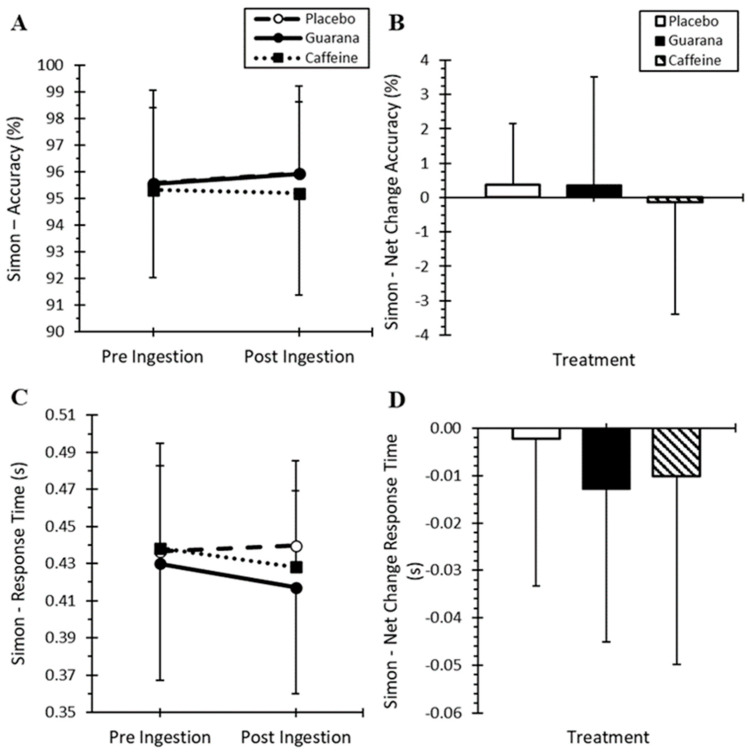
Mean (±SD) Simon Task performance for accuracy (**A**), net change in accuracy (**B**), response time (**C**), and net change in response time (**D**) in all participants (*n* = 20) among treatments (placebo, caffeine, and Guarana).

For all participants (*n* = 20; Figure 2A), there were no differences in the Simon Task ACC for treatment, time, or time-by-treatment interaction (*p* > 0.05), or net change in the Simon Task ACC from pre-ingestion to post-ingestion (Figure 2B). There were no differences for treatment effect (*p* = 0.433), time effect (*p* = 0.066) or time-by-treatment interaction effects (*p* = 0.613) in the Simon Task RT (Figure 2C). No differences were observed among the treatments for net change in the Simon Task RT from pre-ingestion to post-ingestion (Figure 2D).

In the exercise sub-group (*n* = 11), there were no significant treatment differences for net change scores in the Simon Task from baseline, following physical fatigue (cycling test) after ingesting the three supplements ~3 h earlier (*p* = 0.893, Table 1). Although RT became faster relative to baseline, there were no treatment differences due to GUA, CAF, and PLA (*p* = 0.535).

### 3.2. 2N-Back Task Performance

**Figure 3:**
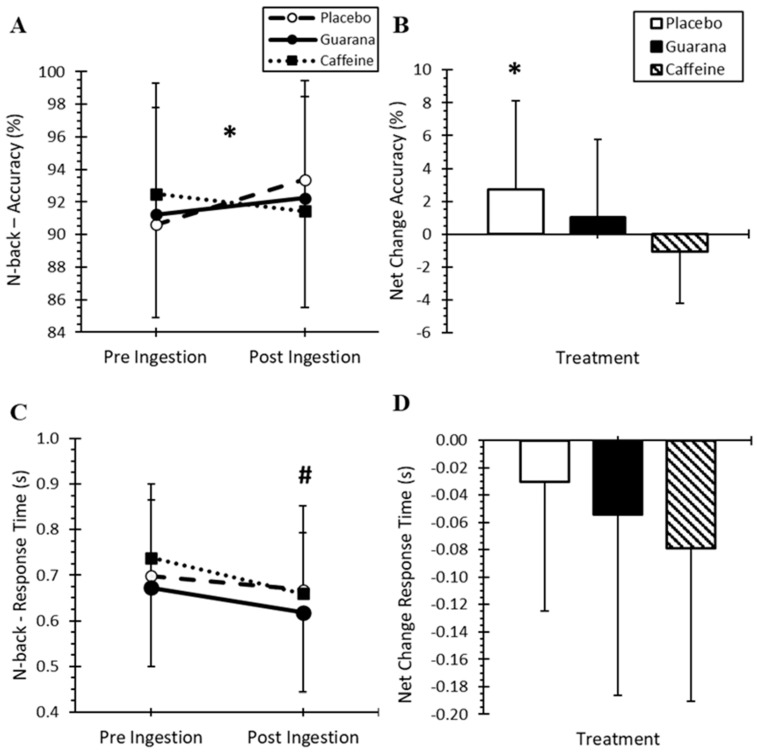
Mean (±SD) 2N-Back Task performance for accuracy (**A**), net change in accuracy (**B**), response time (**C**), and net change in response time (**D**) among treatments. (**A**) * Significantly higher accuracy over time for placebo (*p* = 0.033) and (**B**) * Significantly higher for placebo compared to caffeine (*p* = 0.019). (**C**) # Significantly faster overall time effect (*p* = 0.001).

There were no differences in the 2N-Back ACC (Figure 3A) for treatment (*p* = 0.962) or time effects (*p* = 0.169); however, there was a significant time-by-treatment interaction (*p* = 0.029). For PLA, ACC was higher (*p* = 0.033) at post-ingestion (93.4 ± 6.1%) compared to pre-ingestion (90.6 ± 7.2%). Net change in ACC (Figure 3B) differed by treatments (*p* = 0.029) as PLA (+2.8 ± 5.4%) was improved (*p* = 0.019) versus CAF (−1.1 ± 3.1%). There were no differences in RT among treatments (*p* = 0.086, Figure 3C) or time-by-treatment interaction effects (*p* = 0.438). However, there was an overall time effect for RT (*p* = 0.001) with slower RT pre-ingestion (0.70 ± 0.15 s) compared to post-ingestion (0.65 ± 0.14 s). Net change in RT (Figure 3D) was not different among the treatments.

In the exercise sub-group (*n* = 11), there were no significant treatment differences for net change scores in the 2N-Back Task from baseline to following physical fatigue (cycling test) after ingesting the three supplements ~3 h earlier (*p* = 0.080, Table 1). Although RT became faster relative to baseline, there were no treatment differences due to GUA, CAF and PLA (*p* = 0.963).

### 3.3. NASA Task Load Index Fatigue Scores

**Figure 4:**
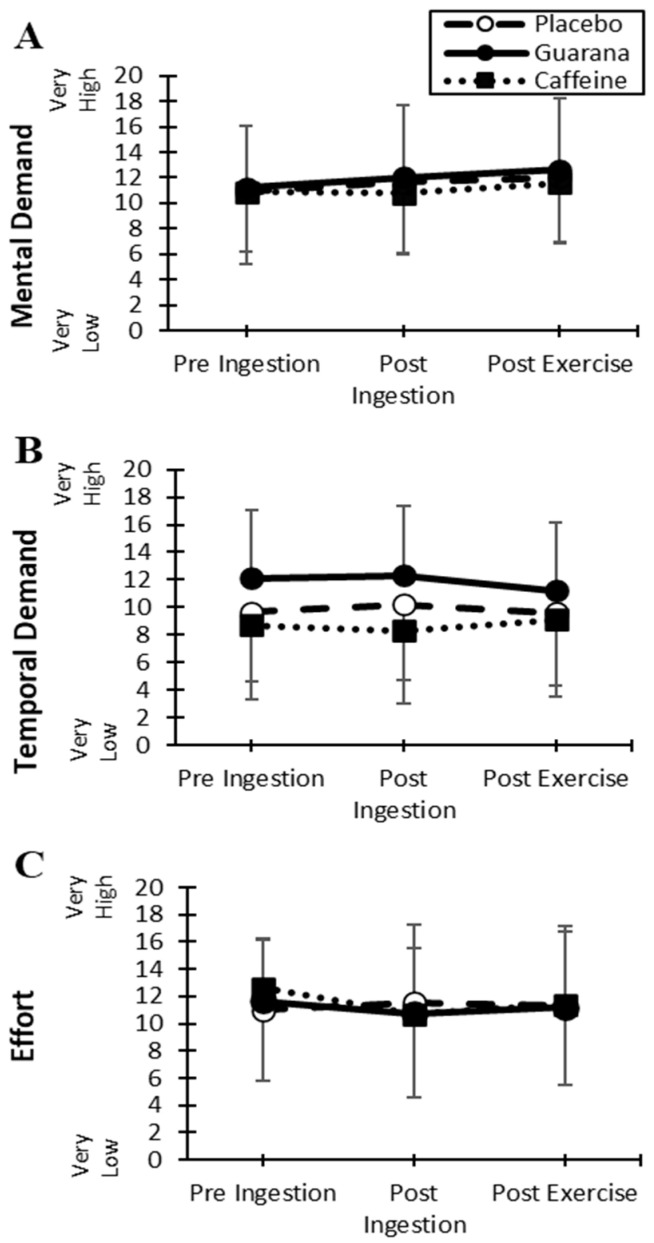
Mean (±SD) NASA Task Load Index for mental demand (**A**), temporal demand (**B**), and effort (**C**) over time among treatments (exercise subgroup *n* = 11).

In the exercise subgroup, there were no significant treatment, time, or time-by-treatment interaction effects on all sub-score ratings (*p* > 0.05), regarding mental demand, physical demand, temporal demand, performance, effort, and frustration. The most closely related sub-scores to the mental workload of cognitive assessments include temporal demand, mental demand, and effort (Figure 4).

### 3.4. Brunel Mood Scale Scores

In the exercise subgroup, there were no significant treatment or time-by-treatment interaction effects for all mood sub-score ratings (*p* > 0.05) for anger, confusion, depression, fatigue, tension, and vigor. There was a significant time effect (*p* = 0.001), but only for fatigue ratings. Post hoc analysis shows that post-ingestion effects were consistently lower than at pre-ingestion and post-exercise effects (*p* = 0.006 and *p* = 0.010, respectively).

### 3.5. Motivation and Energy Visual Analog Ratings

**Figure 5:**
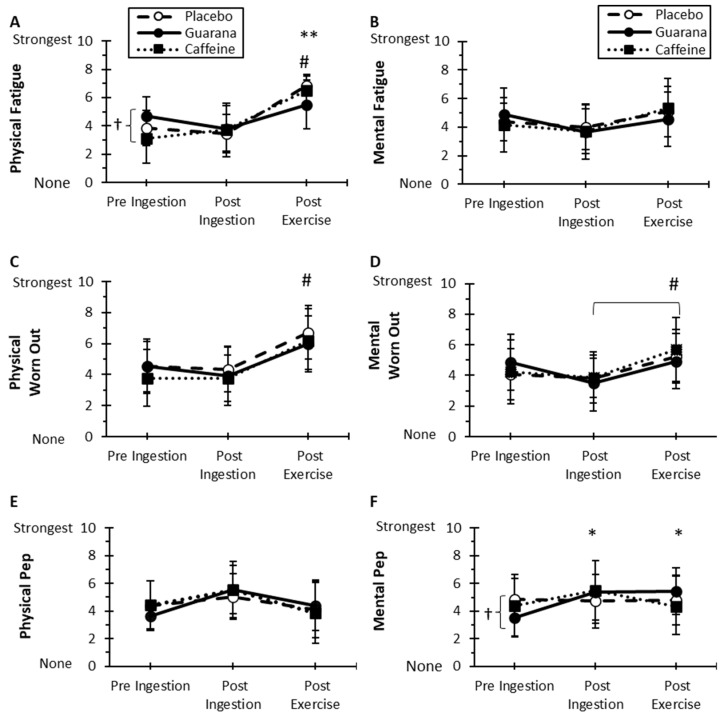
Mean (±SD) energy visual analog ratings for physical (**A**) and mental (**B**) fatigue, physical (**C**) and mentally (**D**) worn out, and physical (**E**) and mental (**F**) pep among treatments in exercise subgroup (*n* = 11). (**A**) # Post-exercise has a higher overall time effect than pre- and post-ingestion (*p* = 0.001 and *p* = 0.002, respectively). † Greater pre-ingestion effects for Guarana versus caffeine (*p* = 0.045). ** Placebo and caffeine have greater post-exercise versus pre-ingestion (*p* = 0.001 and *p* = 0.001, respectively) and post-ingestion effects (*p* = 0.001 and *p* = 0.006, respectively). (**C**) # Post-exercise has a higher overall time effect than pre-ingestion and post-ingestion (*p* = 0.031 and *p* = 0.027, respectively). (**D**) # Post-exercise has a higher overall time effect than post-ingestion (*p* = 0.047). (**F**) † Placebo was greater than Guarana at pre-ingestion (*p* = 0.032). * Post-ingestion (*p* = 0.006) and post-exercise (*p* = 0.018) for Guarana higher than pre-ingestion.

As presented in Figure 5, there were no treatment, time, or time-by-treatment interaction effects for the following: fatigue, energy, vigor, exhaustion, and motivation (*p* > 0.05). For the mentally worn out item, there was only a time effect (*p* = 0.024) with higher post-exercise ratings compared to pre-ingestion ones (*p* = 0.047). There was a treatment-by-time interaction effect (*p* = 0.030) for mental pep (Figure 5F). Pre-ingestion for GUA was lower than pre-ingestion than PLA (*p* = 0.032), which translated into comparatively higher post-ingestion and post-exercise scores (*p* = 0.006 and *p* = 0.018, respectively).

As expected, the time effect for physical fatigue (*p* = 0.001, Figure 5A), mentally worn out (*p* = 0.002, Figure 5C), and exhaustion (*p* = 0.001) had higher post-exercise ratings than the pre-ingestion (*p* = 0.001, *p* = 0.031, and *p* = 0.004, respectively) and post-ingestion levels (*p* = 0.002, *p* = 0.027, and *p* = 0.006, respectively). There were no treatment, time, or time-by-treatment interaction effects for physical pep, energy, and vigor (*p* > 0.05). However, (Figure 5A), there was a treatment-by-time interaction (*p* = 0.001) for physical fatigue. At pre-ingestion, GUA had higher physical fatigue than CAF (*p* = 0.045). Post-exercise ratings for PLA and CAF were higher in physical fatigue than pre-ingestion (*p* = 0.001 and *p* = 0.001, respectively) and post-ingestion ratings (*p* = 0.001 and *p* = 0.006, respectively). For physical exhaustion, there was a treatment-by-time interaction effect (*p* = 0.029) with post-exercise GUA and CAF being higher than pre-ingestion (*p* = 0.044 and *p* = 0.004, respectively) and post-ingestion levels, but for only CAF (*p* = 0.003).

### 3.6. Heart Rate Variability Recordings

There were no significant treatment effects for HRV RMSSD post-ingestion (PLA = 61.2 ± 20.7, GUA = 61.6 ± 19.4, CAF = 62.3 ± 16.8 ms) or post-exercise (PLA = 25.5 ± 12.9, GUA = 23.0 ± 11.9, CAF = 24.5 ± 12.4 ms), nor time-by-treatment interaction (*p* > 0.05). As anticipated, due to the sympathetic activation with exercise, there was an overall time effect with lower RMSSD (*p* < 0.001) post-exercise (24.5 ± 12.4 ms) compared to pre-ingestion (56.9 ± 12.7 ms) and post-ingestion (61.7 ± 19.3 ms).

## 4. Discussion

The results indicate that natural Guarana seed extract did not consistently improve the cognitive or mood domains assessed either alone after ingestion or in combination with physical fatigue. To our knowledge, this is one of the first published investigations comparing the mental performance effects of Guarana to both a placebo control and a low caffeine dose after a fatiguing exercise along with HRV analysis. The only notable GUA supplement effect over time was an improved rating in one mental–visual analog scale (i.e., mental pep) both after ingestion and subsequent physical fatigue (52% and 54% improvement, respectively). However, this may have been due, in part, to differential ratings among the supplements at the baseline (prior to ingestion). Specifically, PLA had 39% higher mental pep ratings compared to GUA at pre-ingestion. As a result, a lower baseline for GUA in this one rating (which was not aligned with other mental fatigue ratings) makes this isolated result difficult to interpret on its own merits. One potential mechanism explaining greater mental alertness and energy from a low dose of caffeine (i.e., sympathetic activation) was not observed for either the CAF or GUA treatments based on HRV. Therefore, whether the lack of cognitive and mood performance differences with GUA is attributed to an insufficient dosage of caffeine remains unresolved.

Reported cognitive benefits due to caffeine are most frequently observed when taken in dosages > 200 mg [16]. Caffeine dosage at this minimum threshold or above has the potential to enhance memory consolidation [21], vigilance performance [22], ratings of alertness [22], and speed of encoding new information [22]. Despite the paucity of such research, claims have been made that lower doses of caffeine may also enhance performance, specifically on attention tasks compared to higher doses > 200 mg [17]. In summary, the dose–response effect on cognitive performance with caffeine remains uncertain [16]. Therefore, this study originally attempted to match the low caffeine dosage listed on the commercially available Guarana supplements at 100 mg to the same dose of caffeine alone. Unfortunately, the manufacturer’s label incorrectly stated the caffeine content for GUA capsules (listed as 100 mg, later confirmed as 130 mg by NSF independent analysis). This discrepancy might be influenced by variable amounts of caffeine found in Guarana. Guarana seeds typically have 4–8% caffeine but that of Guarana extract may be higher (~20%) than coffee beans [18,19,20].

To induce a cognitive and mood effect with caffeine, several additional covariates must be considered, including participants’ daily consumption habits, age, gender, body composition, disease, and genetic influences on the pharmacokinetics of caffeine [13,14]. These factors may influence how participants respond to caffeine, which could differentially modify the effect caffeine may have on various individuals [14]. Caffeine tends to significantly improve the cognitive performance of habitual caffeine consumers under acute ingestion for spatial memory tasks and rapid visual information processing tasks compared to non-habitual caffeine drinkers [15]. The habitual caffeine intake was not recorded for all participants; however, no participants in the exercise subgroup had daily consumption above an average caffeine intake (~250 mg per day).

This present study failed to align with some research findings that the benefits obtained after consuming Guarana are associated with greater cognitive performance improvements than the caffeine content alone might suggest [43]. An earlier meta-analysis [27] from our group indicated that acute Guarana ingestion may influence specific response time outcomes with a small magnitude of effect, but not the accuracy in various cognitive performance assessments. In contrast, the present study found no difference in RT among treatments in cognitive measures involving attention and working memory. Although there was a positive net change in ACC in one task (2N-Back) for PLA versus CAF, this appears to be driven primarily by three participants with apparently large improvements over time (10–11%). This suggests a potential task-learning effect among these participants that cannot be attributed to a treatment effect. No net change in ACC for the Simon Task was observed for any treatments, as approximately 95% of responses were correct, indicating that this task had a ceiling effect and likely lacked the sensitivity to produce observable differences among treatments.

In this present study, the lack of discernible differences between treatments in the Simon and 2N-Back Tasks indicates that GUA had no beneficial effects on the respective response inhibition and working memory parameters assessed under either the normal or physically fatigued states. This conflicts with other individual studies compiled earlier [27] which found that a single dose of 37.5 mg and 75 mg Guarana increased the secondary memory accuracy of activities in Cognitive Drug Research (CDR) Computerized Assessment Battery among healthy young adults [28]. In relation to memory improvements, another study [29] also found that participants who took 75 mg of Guarana displayed improved scores in secondary memory activities of the CDR Computerized Assessment Battery at 150 min after ingestion compared to the placebo. In the same study, improvements in accuracy for other working memory parameters assessed by Serial Sevens Subtraction Task were found with 75 mg Guarana at 360 min after ingestion compared to the placebo [29]. However, a few studies [34,44] have also supported that Guarana ingestion does not always have a positive impact on cognitive performance, which is more aligned with our present study results. In healthy young adults, it was observed that 222 mg of Guarana in a vitamin and mineral complex had no treatment differences in response time measured by the Choice Reaction Time Task at 30, 75, and 150 min after ingestion compared to a placebo [34]. In the same study, no treatment differences were found in the working memory accuracy and response time parameters measured by Delayed Word Recall Tasks at 30, 75, and 150 min after ingestion compared to a placebo [34]. In another study, it was observed that the same 222 mg of Guarana in a vitamin and mineral complex had no between-treatment differences in score and accuracy for working memory parameters measured by the Serial Threes and Sevens Subtraction Tasks up to 80 min after ingestion compared to a placebo [44].

According to other studies, several elements of mood and fatigue perception may be positively impacted following acute Guarana ingestion [28,45]. A single dose of 300 mg Guarana increased alertness among young adults in the Bond–Lader Mood Assessment [28]. Similarly, another study found that a single 100 mg dose of Guarana dissolved in decaffeinated coffee improved alertness ratings 150 min after ingestion in young women, as assessed by the Eighteen Bipolar Visual Analogue Scale [45]. In contrast, male adults ingesting a single dose of 222 mg Guarana had no treatment differences in alertness, calmness, and content ratings in the Bond–Lader Mood Assessment up to 150 min after ingestion [34], which is in alignment with the present study. No difference among treatments was found in Brunel Mood Scale 60 min following ingestion and exercise. Another study examining acute mental fatigue following the ingestion of 222 mg GUA reported reduced mental fatigue ratings in healthy young volunteers up to an hour post-ingestion compared to the placebo [44]. In contrast, the present study lacked any significant treatment differences for mental demand and effort measured by the NASA Task Load and Motivation and Energy Visual Analog Scales. Only in GUA were there improved feelings of mental pep after ingestion and physical fatigue in the exercise subgroup. However, there was no impact on the other corroborating survey items for mental fatigue with GUA. This lack of mental fatigue benefits aligns with another study that found that 222 mg GUA in a vitamin and mineral complex lacked any treatment differences in concentration and mental stamina ratings as measured by an energy visual analog scale [34].

This present study has reported no treatment differences for HRV at various time points after ingestion and exercise. This is in conflict with another study that reported that 300 mg GUA in healthy caffeine consumers had a lesser impact on HRV compared to a significantly lower HRV for CAF and PLA 60–90 min after ingestion [25]. However, we observed an expected time effect for all treatments post-exercise with lower HRV compared to pre-ingestion and post-ingestion. This time effect was similar to other studies [46,47], noting that the reduced HRV after exercise due to changes in vagal tone exerting an influence on heart rate. Activation of the sympathetic nervous system to meet the physiological demand of exercise usually correlates with a decrease from the baseline HRV levels after exercise [42]. Therefore, the lack of differences between GUA and CAF compared to PLA suggests that these treatments were insufficient to impact the autonomic nervous system response to physical exercise.

Lastly, other studies have suggested potential benefits due to other physiological mechanisms involving additional bioactive compounds found in Guarana [24,48,49,50]. Unlike other caffeinated products, Guarana also naturally contains flavonoids (e.g., catechins and epicatechins) and proanthocyanidins [24]. Flavonoids are proposed to be strong antioxidants, scavenging and neutralizing dangerous reactive oxygen species and free radicals in the brain [48]. This is significant because neuronal cell damage brought on by oxidative stress has been linked to cognitive decline and neurodegenerative disorders [48]. Flavonoids shield neurons and support general cognitive function by lowering oxidative stress [49]. Findings from a small selection of studies with randomized, controlled, human trials suggest a positive correlation between flavonoid consumption and cognitive function [49]. Proanthocyanidins can control the activity of inflammatory pathways and have anti-inflammatory effects, maintaining cognitive function by decreasing neuroinflammation [50]. However, additional comprehensive investigations are required to link these findings and their application to cognitive and mood benefits based on acute Guarana ingestion.

The inconsistency in findings may be attributable to differences in research design, supplement doses, GUA sources, participants, and the particular cognitive and mood tasks performed. These contradictory findings point to the need for additional research to determine the cognitive and mood effects associated with Guarana on working memory, attention, response inhibition, and mental fatigue. There are several limitations in the current study. As a result of using convenience sampling, there were only three females recruited, making it impractical to examine sex-related differences. Although our sample size was typical for CAF studies [51], it may have been underpowered to detect differences across cognitive and mood outcome measures. Due to the lack of access to equipment and survey/scales not being translated to Portuguese, only certain assessments were recorded in a separate international laboratory for the non-exercise subgroup.

## 5. Conclusions

Novel findings of this present study indicate that Guarana seed extract did not consistently improve cognitive and mood domains following acute ingestion in comparison to either a low dose (100 mg) of caffeine or placebo. This suggests Guarana seed extract (500 mg) with a naturally low dose of caffeine (≤130 mg) cannot be categorized as a mood- or cognitive-enhancing supplement. Secondly, we also found limited mental performance benefits with a low caffeine dose for individuals who have a below-average habitual caffeine intake. Following physical fatigue, this non-significant result persisted although there was higher mental pep observed with Guarana, which appears unrelated to changes in vagal modulation. Whether Guarana seed extract confers additional mental related benefits beyond its caffeine content cannot be ascertained, indicating that additional investigations are warranted.

## Figures and Tables

**Table 1:** Net change (±SD) in ACC and RT (Post Exercise vs. Pre-Ingestion) for the Simon and 2N-Back Tasks in the exercise subgroup (*n* = 11) for placebo, caffeine, and Guarana.

	Placebo	Guarana	Caffeine
∆Simon ACC (%)	−0.57 ± 1.71	−0.23 ± 2.61	−0.57 ± 3.46
∆Simon RT (s)	−0.02 ± 0.03	−0.03 ± 0.02	−0.04 ± 0.06
∆2N-Back ACC (%)	1.93 ± 2.12	1.82 ± 3.18	−0.68 ± 3.89
∆2N-Back RT (s)	−0.10 ± 0.10	−0.10 ± 0.10	−0.09 ± 0.15

## Data Availability

Data are unavailable due to privacy restrictions across the two institutions in two countries.

## References

[1] Harvey P.D. (2019). Domains of cognition and their assessment. Dialogues Clin. Neurosci..

[2] Tyng C.M., Amin H.U., Saad M.N.M., Malik A.S. (2017). The influences of emotion on learning and memory. Front. Psychol..

[3] Tran Y., Craig A., Craig R., Chai R., Nguyen H.T. (2020). The influence of mental fatigue on brain activity: Evidence from a systematic review with meta-analyses. Psychophysiology.

[4] Hakim H., Khemiri A., Chortane O.G., Boukari S., Chortane S.G., Bianco A., Marsigliante S., Patti A., Muscella A. (2022). Mental fatigue effects on the produced perception of effort and its impact on subsequent physical performances. Int. J. Environ. Res. Public Health.

[5] Madireddy S. (2021). Most effective combination of nutraceuticals for improved memory and cognitive performance in the house cricket, Acheta domesticus. Nutrients.

[6] Childs E. (2014). Influence of energy drink ingredients on mood and cognitive performance. Nutr. Rev..

[7] Frary C.D., Johnson R.K., Wang M.Q. (2005). Food sources and intakes of caffeine in the diets of persons in the United States. J. Am. Diet. Assoc..

[8] Spaeth A.M., Goel N., Dinges D.F. (2014). Cumulative neurobehavioral and physiological effects of chronic caffeine intake: Individual differences and implications for the use of caffeinated energy products. Nutr. Rev..

[9] Hung N., Akhter S., Miao Y. (2021). Pathways and mechanism of caffeine binding to human adenosine A2A receptor. Front. Mol. Biosci..

[10] Fiani B., Zhu L., Musch B., Briceno S., Andel R., Sadeq N., Ansari A.Z. (2021). The neurophysiology of caffeine as a central nervous system stimulant and the resultant effects on cognitive function. Cureus.

[11] Voskoboinik A., Koh Y., Kistler P.M. (2019). Cardiovascular effects of caffeinated beverages. Trends Cardiovasc. Med..

[12] Grgić J., Del Coso J. (2021). Ergogenic effects of acute caffeine intake on muscular endurance and muscular strength in women: A meta-analysis. Int. J. Environ. Res. Public Health.

[13] Nehlig A. (2018). Interindividual differences in caffeine metabolism and factors driving caffeine consumption. Pharmacol. Rev..

[14] Yang A.H., Palmer A.A., de Wit H. (2010). Genetics of caffeine consumption and responses to caffeine. Psychopharmacology.

[15] Haskell C.F., Kennedy D.O., Wesnes K., Scholey A.B. (2005). Cognitive and mood improvements of caffeine in habitual consumers and habitual non-consumers of caffeine. Psychopharmacology.

[16] Zhang R., Madan C.R. (2021). How does caffeine influence memory? Drug, experimental, and demographic factors. Neurosci. Biobehav. Rev..

[17] Bryant C., Farmer A., Tiplady B., Keating J., Sherwood R., Swift C.G., Jackson S.H.D. (1998). Psychomotor performance: Investigating the dose-response relationship for caffeine and theophylline in elderly volunteers. Eur. J. Clin. Pharmacol..

[18] Torres E.B., Pinaffi-Langley A.C.C., De Souza Figueira M., Cordeiro K.S., Negrão L.D., Soares M.J., da Silva C.P., Alfino M.C.Z., Sampaio G.R., de Camargo A.C. (2021). Effects of the consumption of guarana on human health: A narrative review. Compr. Rev. Food Sci. Food Saf..

[19] Schimpl F.C., Da Silva J.A.G., De Carvalho Gonçalves J.F., Mazzafera P. (2013). Guarana: Revisiting a highly caffeinated plant from the Amazon. J. Ethnopharmacol..

[20] Olechno E., Puścion-Jakubik A., Zujko M.E., Socha K. (2021). Influence of various factors on caffeine content in coffee brews. Foods.

[21] Borota D., Murray E., Keceli G., Chang A., Watabe J.M., Ly M., Toscano J.P., Yassa M.A. (2014). Post-study caffeine administration enhances memory consolidation in humans. Nat. Neurosci..

[22] Smith A., Sutherland D.E., Christopher G. (2005). Effects of repeated doses of caffeine on mood and performance of alert and fatigued volunteers. J. Psychopharmacol..

[23] Da Silva G.S., Canuto K.M., Ribeiro P.R.V., de Brito E.S., Nascimento M.M., Zocolo G.J., de Jesus R.M. (2017). Chemical profiling of guarana seeds (*Paullinia cupana*) from different geographical origins using UPLC-QTOF-MS combined with chemometrics. Food Res. Int..

[24] Silva C.A., Sampaio G.R., Freitas R.A.M.S., Da Silva Torres E.A.F. (2018). Polyphenols from guaraná after in vitro digestion: Evaluation of bioaccessibility and inhibition of activity of carbohydrate-hydrolyzing enzymes. Food Chem..

[25] Pomportes L., Davranche K., Brisswalter I., Hays A., Brisswalter J. (2014). Heart rate variability and cognitive function following a multi-vitamin and mineral supplementation with added guarana (*Paullinia cupana*). Nutrients.

[26] Pomportes L., Brisswalter J., Hays A., Davranche K. (2019). Effects of carbohydrate, caffeine, and guarana on cognitive performance, perceived exertion, and shooting performance in high-level athletes. Int. J. Sports Physiol. Perform..

[27] Hack B., Penna E.M., Talik T., Chandrashekhar R., Millard-Stafford M.L. (2023). Effect of guarana (*Paullinia cupana*) on cognitive performance: A systematic review and meta-analysis. Nutrients.

[28] Haskell C.F., Kennedy D.O., Wesnes K., Milne A., Scholey A.B. (2006). A double-blind, placebo-controlled, multi-dose evaluation of the acute behavioural effects of guaraná in humans. J. Psychopharmacol..

[29] Kennedy D.O., Haskell C.F., Wesnes K., Scholey A.B. (2004). Improved cognitive performance in human volunteers following administration of guarana (*Paullinia cupana*) extract: Comparison and interaction with Panax ginseng. Pharmacol. Biochem. Behav..

[30] Staiano W., Bonet L.R.S., Romagnoli M., Ring C. (2023). Mental fatigue impairs repeated sprint and jump performance in team sport athletes. J. Sci. Med. Sport.

[31] Van Cutsem J., Marcora S.M., De Pauw K., Bailey S., Meeusen R., Roelands B. (2017). The effects of mental fatigue on physical performance: A systematic review. Sports Med..

[32] Grgić J., Grgic I., Pickering C., Schoenfeld B.J., Bishop D.J., Pedisic Z. (2019). Wake up and smell the coffee: Caffeine supplementation and exercise performance—An umbrella review of 21 published meta-analyses. Br. J. Sports Med..

[33] Gurney T., Bradley N., Izquierdo D., Ronca F. (2022). Cognitive effects of guarana supplementation with maximal intensity cycling. Br. J. Nutr..

[34] Veasey R.C., Haskell-Ramsay C.F., Kennedy D.N., Wishart K., Maggini S., Fuchs C.J., Stevenson E.J. (2015). The effects of supplementation with a vitamin and mineral complex with guaraná prior to fasted exercise on affect, exertion, cognitive performance, and substrate metabolism: A randomized controlled trial. Nutrients.

[35] Penna E.M., Harp A., Hack B., Talik T.N., Millard-Stafford M. (2024). Guarana (*Paullinia cupana*) but not low-dose caffeine improves cycling time-trial performance versus placebo. Int. J. Sport Nutr. Exerc. Metab..

[36] Simon J., Rudell A.P. (1967). Auditory S-R compatibility: The effect of an irrelevant cue on information processing. J. Appl. Psychol..

[37] Kirchner W.K. (1958). Age differences in short-term retention of rapidly changing information. J. Exp. Psychol..

[38] Terry P.C., Lane A.M. (2010). User Guide to Brunel Mood Scale.

[39] Hart S.G., Staveland L.E. (1988). Development of NASA-TLX (Task Load Index): Results of empirical and theoretical research. Adv. Psychol..

[40] Maridakis V., O’Connor P.J., Tomporowski P.D. (2009). Sensitivity to change in cognitive performance and mood measures of energy and fatigue in response to morning caffeine alone or in combination with carbohydrate. Int. J. Neurosci..

[41] Himariotis A.T., Coffey K.F., Noël S., Cornell D.J. (2022). Validity of a smartphone application in calculating measures of heart rate variability. Sensors.

[42] Shaffer F., Ginsberg J.P. (2017). An overview of heart rate variability metrics and norms. Front. Public Health.

[43] Kennedy D.N., Wightman E.L. (2022). Mental performance and sport: Caffeine and co-consumed bioactive ingredients. Sports Med..

[44] Kennedy D.O., Haskell C.F., Robertson B., Reay J., Brewster-Maund C., Luedemann J., Scholey A.B. (2008). Improved cognitive performance and mental fatigue following a multi-vitamin and mineral supplement with added guaraná (*Paullinia cupana*). Appetite.

[45] Meyer K., Ball P. (2004). Psychological and cardiovascular effects of guarana and yerba mate: A comparison with coffee. Interam. J. Psychol..

[46] Michael S., Graham K., Davis G.M. (2017). Cardiac autonomic responses during exercise and post-exercise recovery using heart rate variability and systolic time intervals—A review. Front. Physiol..

[47] Mongin D., Chabert C., Extremera M.G., Hue O., Courvoisier D.S., Carpena P., Galvan P.A.B. (2022). Decrease of heart rate variability during exercise: An index of cardiorespiratory fitness. PLoS ONE.

[48] Spencer J.P.E. (2009). Flavonoids and brain health: Multiple effects underpinned by common mechanisms. Genes Nutr..

[49] Macready A.L., Kennedy O.B., Ellis J.A., Williams C.M., Spencer J.P.E., Butler L.T. (2009). Flavonoids and cognitive function: A review of human randomized controlled trial studies and recommendations for future studies. Genes Nutr..

[50] Yang L., Xian D., Xiong X., Lai R., Song J., Zhong J. (2018). Proanthocyanidins against oxidative stress: From molecular mechanisms to clinical applications. BioMed Res. Int..

[51] Conger S.A., Tuthill L.M., Millard-Stafford M. (2023). Does caffeine increase fat metabolism? A systematic review and meta-analysis. Int. J. Sport Nutr. Exerc. Metab..

